# Effect of Surface Modification of Cu Electrodes by Ag Nanoparticle Spray Coating on the Products and Electrolytic Potential of Electrochemical CO_2_ Reduction

**DOI:** 10.3390/molecules31162803

**Published:** 2026-08-12

**Authors:** Kazuki Koike, Takeharu Murakami, Kentaro Inoue, Takayo Ogawa, Katsushi Fujii, Satoshi Wada, Atsushi Ogura

**Affiliations:** 1 School of Science and Technology, Meiji University, 1-1-1 Higashi-Mita, Tama-ku, Kawasaki 214-8571, Kanagawa, Japan; meijikenino@gmail.com (K.I.); a_ogura@meiji.ac.jp (A.O.); 2 RIKEN RAP, 2-1 Hirosawa, Wako 351-0198, Saitama, Japan; takeharu.murakami@riken.jp (T.M.); pogawa@riken.jp (T.O.); katsushi.fujii@riken.jp (K.F.); swada@riken.jp (S.W.); 3 Meiji Renewable Energy Laboratory, Kawasaki 214-8571, Kanagawa, Japan

**Keywords:** electrochemical CO_2_ reduction, Ag nanoparticle, products selectivity, Cu electrode surface modification

## Abstract

Electrochemical CO_2_ reduction reaction (eCO_2_RR) is a promising technology for carbon utilization, yet achieving high product selectivity and long-term stability remains a critical challenge. In this study, we investigated the performance and surface stability of Cu electrodes modified with Ag nanoparticles using a spray-coating method. While a bare Cu reference electrode exhibited an initial starting period dominated by hydrogen evolution before shifting toward hydrocarbon production after two hours, the Ag-spray-coated Cu electrode demonstrated immediate and stable catalytic activity. Electrode potential remained stable throughout the 12 h evaluation, in contrast to the negative shifts observed with the bare Cu electrode. Ambient pressure hard X-ray photoelectron spectroscopy (AP-HAXPES) revealed that while the bare Cu surface remained metallic, the Ag-spray-coated Cu surface existed as Cu_2_O during the reaction. The enhanced selectivity and stability are attributed to a spillover mechanism, where CO generated on the Ag nanoparticles migrates to adjacent Cu_2_O sites, inhibiting hydrogen evolution and facilitating efficient reduction to methane and ethylene from the onset of electrolysis. These findings demonstrate that surface modification via nanoparticle spray coating is a highly effective strategy for achieving selective and stable CO_2_ conversion on bimetallic catalysts.

## 1. Introduction

Global warming has emerged as a critical international crisis in recent decades, primarily attributed to greenhouse gas emissions from anthropogenic activities, such as fossil fuel combustion. Reducing CO_2_ emissions, which is the dominant greenhouse gas, has become a very difficult global challenge, as underscored by the Paris Agreement. This agreement aims to limit the average global temperature increase to well below 2 °C, and preferably to 1.5 °C, compared to pre-industrial levels [1,2]. However, current national emission reduction targets are projected to be insufficient, potentially leading to a temperature rise exceeding 2 °C by the end of the 21st century [3]. Consequently, more aggressive CO_2_ mitigation strategies are urgently required worldwide. While transitioning from fossil fuels to carbon-neutral energy sources is essential, these efforts primarily prevent new emissions rather than addressing CO_2_ already present in the atmosphere. To mitigate existing CO_2_ reduction, Carbon Capture and Storage (CCS) technologies have been developed. However, the scarcity of suitable storage sites remains a significant bottleneck. In response, Carbon Capture and Utilization (CCU), which treats CO_2_ as a valuable resource, is gaining significant attention. Within the scope of CCU, processes such as CO_2_ methanation, which convert CO_2_ into CH_4_ via reaction with H_2_, are being extensively researched [4].

To actively reduce atmospheric CO_2_ concentrations, the electrochemical CO_2_ reduction reaction (eCO_2_RR) powered by renewable energy sources, such as solar and wind power, has been proposed. It is well-established that eCO_2_RR using copper (Cu) electrodes can yield a variety of products, including H_2_, C_2_H_4_, CH_4_, CO, HCOOH, and C_2_H_5_OH [5,6]. For the practical application of eCO_2_RR, it is essential to selectively produce industrially valuable feedstocks such as C_2_H_4_, CH_4_, and C_2_H_5_OH; however, achieving high selectivity remains a challenge. The product distribution of eCO_2_RR is highly dependent on the cathode material and the applied potential. Previous studies have confirmed that silver (Ag) exhibits high catalytic activity for the conversion of CO_2_ to CO [7], while Cu is effective in further reducing CO to CH_4_ and C_2_H_4_ [8,9]. Consequently, research has focused on bimetallic electrodes combining these two metals to enhance the selective production of CH_4_ and C_2_H_4_. Various strategies have been explored to improve selectivity, including Cu-based alloying [10,11,12,13], the fabrication of tandem structures [14], and the development of microstructures via electrolytic reactions [15,16]. Metal modification and interface engineering have been widely explored in both electrocatalytic and photocatalytic systems as practical approaches to influence CO_2_ activation, assist intermediate transformations, and adjust product selectivity [17,18,19].

Furthermore, recent comprehensive reviews have underscored that transitioning from microscopic catalyst engineering to advanced macroscopic electrode architecture design is crucial for optimizing gas–liquid equilibrium and overcoming interfacial mass transfer limitations [20]. Concurrently, strategic focus has been directed toward stabilizing the effective copper valence states (${\mathrm{Cu}}^{\delta +}$) and mitigating surface restructuring under intense operational reducing conditions to achieve industrial-grade longevity [21]. In this study, we evaluate the product selectivity and stability of eCO_2_RR using a Cu electrode modified with Ag via a facile nanoparticle spray-coating method. While previous works rely on pre-engineered, static nanostructures to artificially fix the oxide phases [14,22,23,24], the real-time dynamic evolution of a simple, non-templated spray-deposited metallic interface under operational conditions remains poorly understood. Here, we focus on capturing the dynamic robustness and self-stabilization behaviors of this easily accessible interface.

## 2. Results

### 2.1. Bare Cu Electrode

**Figure 1:**
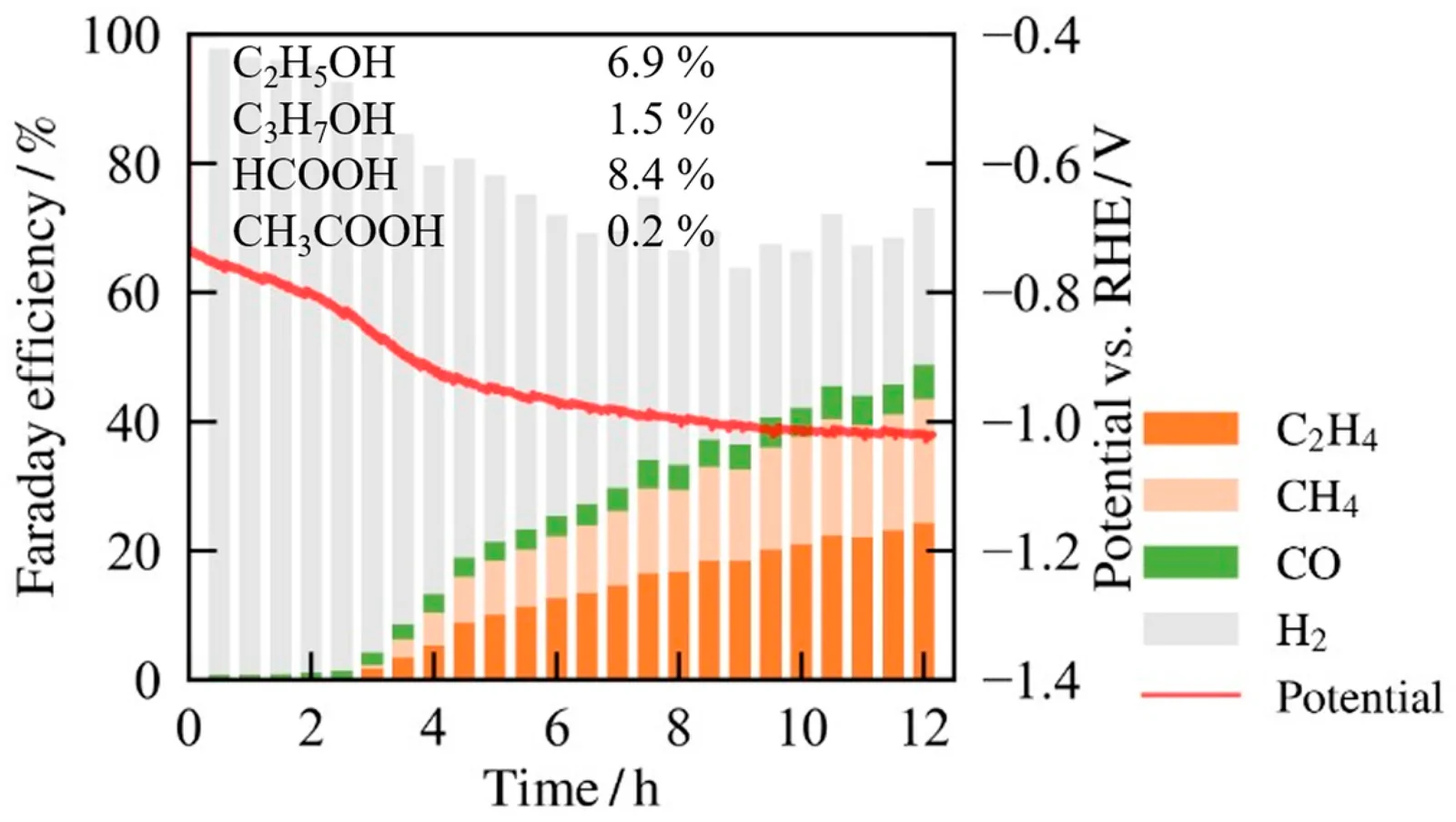
Faraday efficiencies of gaseous products and electrode potential during 12 h of CO_2_ reduction on the bare Cu electrode at a constant current density of −5 mA/cm^2^ in CO_2_-saturated 0.1 M KHCO_3_. The red line denotes the electrode potential (vs. RHE), which has been corrected for ohmic resistance. Numerical values indicate the Faraday efficiencies of liquid products (C_2_H_5_OH, C_3_H_7_OH, HCOOH, and CH_3_COOH) obtained after the 12 h period.

Figure 1 shows the products of CO_2_ reduction with the bare Cu electrode and the potential during electrolysis. Immediately after the reaction starts, only hydrogen (H_2_) generation occurs with a Faradaic efficiency (FE) of nearly 100%. After about two hours, CO_2_ reduction becomes active, and the FE of H_2_ gradually decreases to approximately 25% by the end of the 12 h period. At 12 h, gaseous products include ethylene (C_2_H_4_) with an FE of approximately 25%, methane (CH_4_) at 18%, and carbon monoxide (CO) at 5%. Additionally, liquid products were obtained with FEs of 6.9% for ethanol (C_2_H_5_OH), 1.5% for propanol (C_3_H_7_OH), 8.4% for formic acid (HCOOH), and 0.2% for acetic acid (CH_3_COOH) after the 12 h reaction. Over the full 12 h span, the time-averaged FEs for H_2_, CH_4_, and C_2_H_4_ were approximately 45.0%, 10.5%, and 14.0%, which correspond to average partial current densities of −2.25, −0.53, and −0.70 mA/cm^2^ respectively.

This result explains the time-dependent voltage change under constant current operation because the electrochemical CO_2_ reduction reaction (eCO_2_RR) tends to require a more negative potential compared with the hydrogen evolution reaction (HER). The potential of the electrode during the reaction decreases from approximately −0.74 V to −1.04 V (vs. RHE) as the reaction changes from a state where only H_2_ generation occurs to a state where CO_2_ reduction becomes active. This significant potential drift should be understood as a dynamic surface activation process and a prolonged induction period. As the electrode surface is gradually covered by generated CO intermediates, the hydrogen evolution reaction (HER) is progressively suppressed, forcing the system to thermodynamically adjust its operational overpotential to drive the more complex multi-electron CO_2_ reduction pathway.

**Figure 2:**
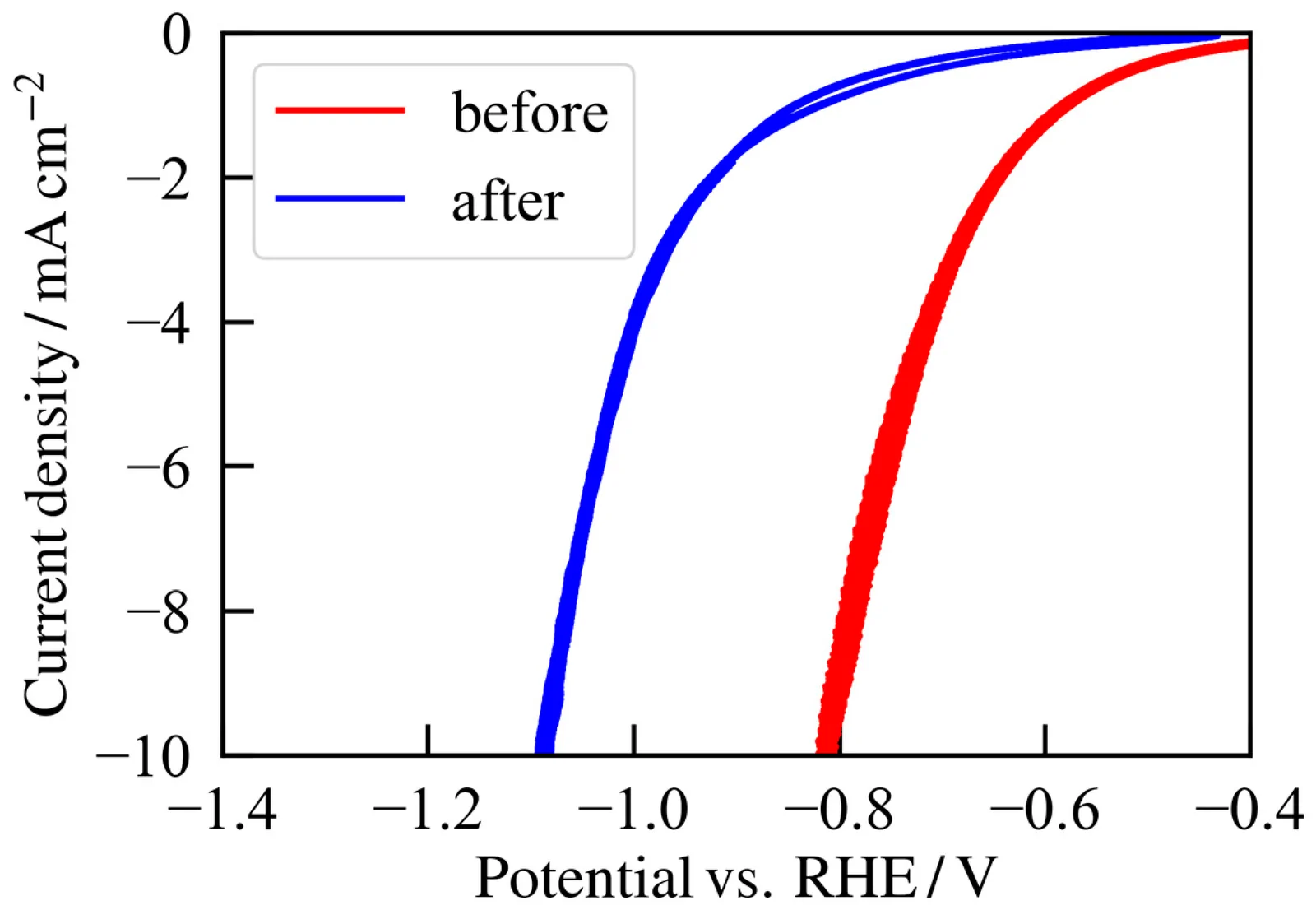
I-V characteristics of the bare Cu electrode measured before (red line) and after (blue line) 12 h of electrolysis in CO_2_-saturated 0.1 M KHCO_3_. All potentials were corrected for ohmic resistance (I-R drop) based on EIS measurements. The significant negative shift in the current onset potential after electrolysis reflects the increased energy requirement as the reaction progresses from hydrogen evolution to CO_2_ reduction.

Figure 2 shows the I-V characteristics of the Cu electrode before and after 12 h of electrolysis. The voltage at which the current begins to rise shifts significantly to more negative values after the electrolysis compared to the initial state. Specifically, the potential required to reach a current density of −10 mA/cm^2^ shifted significantly from approximately −0.81 V to −1.09 V vs. RHE after electrolysis (Figure 2). This marked increase in overpotential reflects the structural and interfacial alterations occurring on the bare Cu surface during long-term operation. Rather than a simple change in thermodynamic demand, this negative shift likely originates from a combination of underlying factors, including potential catalyst poisoning by strongly adsorbed intermediates or trace impurities, irreversible cathodic surface restructuring, and deteriorated interfacial mass transport induced by local pH shifts and gas bubble accumulation.

**Figure 3:**
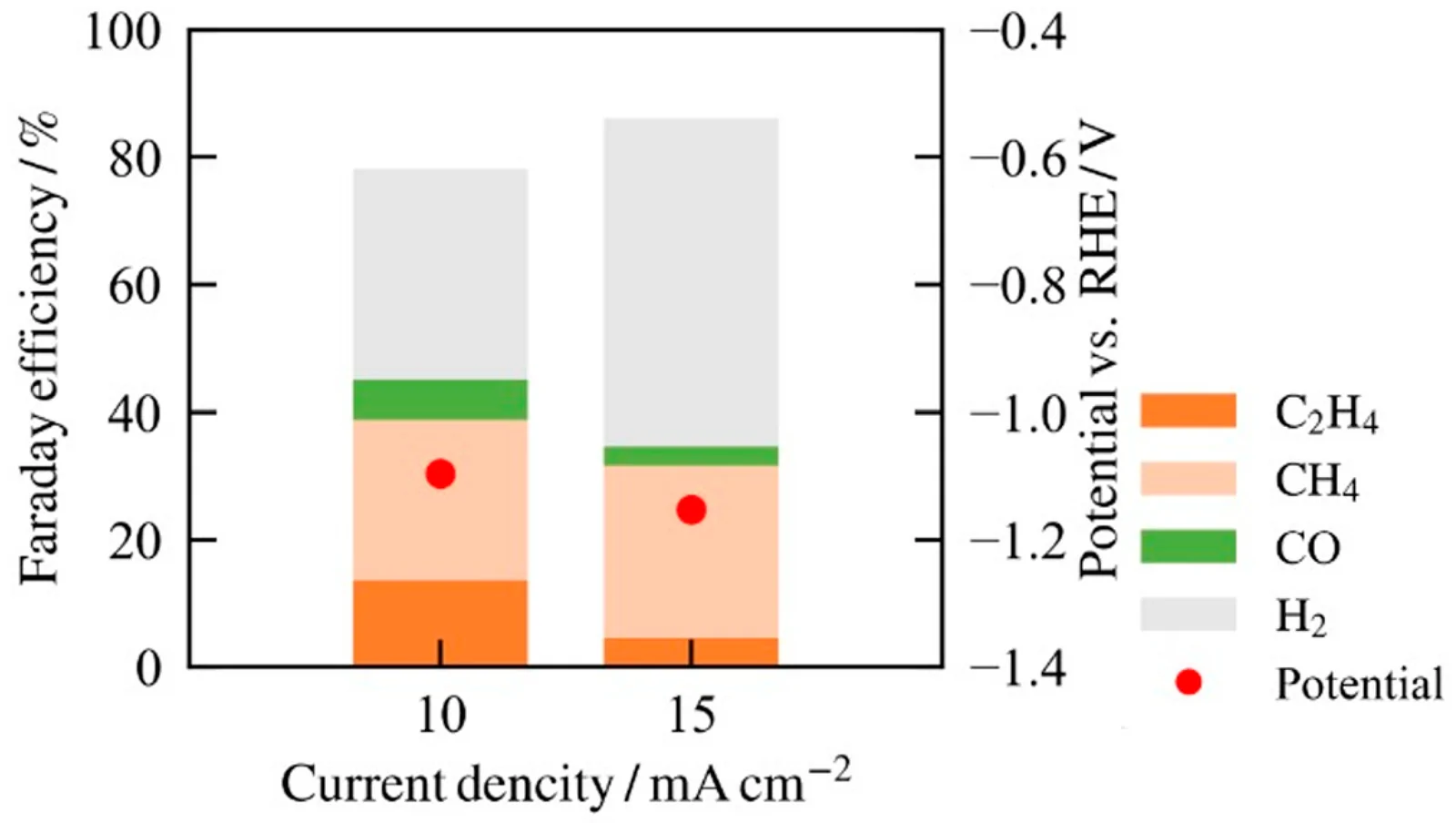
Faradaic efficiencies of gaseous products and operational electrode potentials (vs. RHE) of the pre-conditioned bare Cu electrode during subsequent constant-current electrolysis at high current densities of −10 mA/cm^2^ and −15 mA/cm^2^ in CO_2_-saturated 0.1 M KHCO_3_.

To investigate the operational behavior under higher electrical workloads, further bulk electrolysis was performed on the pre-conditioned bare Cu electrode at increased current densities of −10 mA/cm^2^ and −15 mA/cm^2^ (Figure 3). As the applied current density was increased, the operational potential of the bare Cu electrode shifted to more negative values, stabilizing at −1.10 V vs. RHE under −10 mA/cm^2^ and −1.15 V vs. RHE under −15 mA/cm^2^ (Figure 3). Along with this potential shift, the product selectivity adjusted significantly (Figure 3); the Faradaic efficiency (FE) of H_2_ increased to 33% at −10 mA/cm^2^ and reached 51% at −15 mA/cm^2^, while the FEs for CH_4_ and C_2_H_4_ were determined to be 25% and 14% at −10 mA/cm^2^, and 27% and 4.7% at −15 mA/cm^2^, respectively.

### 2.2. Ag-Spray-Coated Cu Electrode

**Figure 4:**
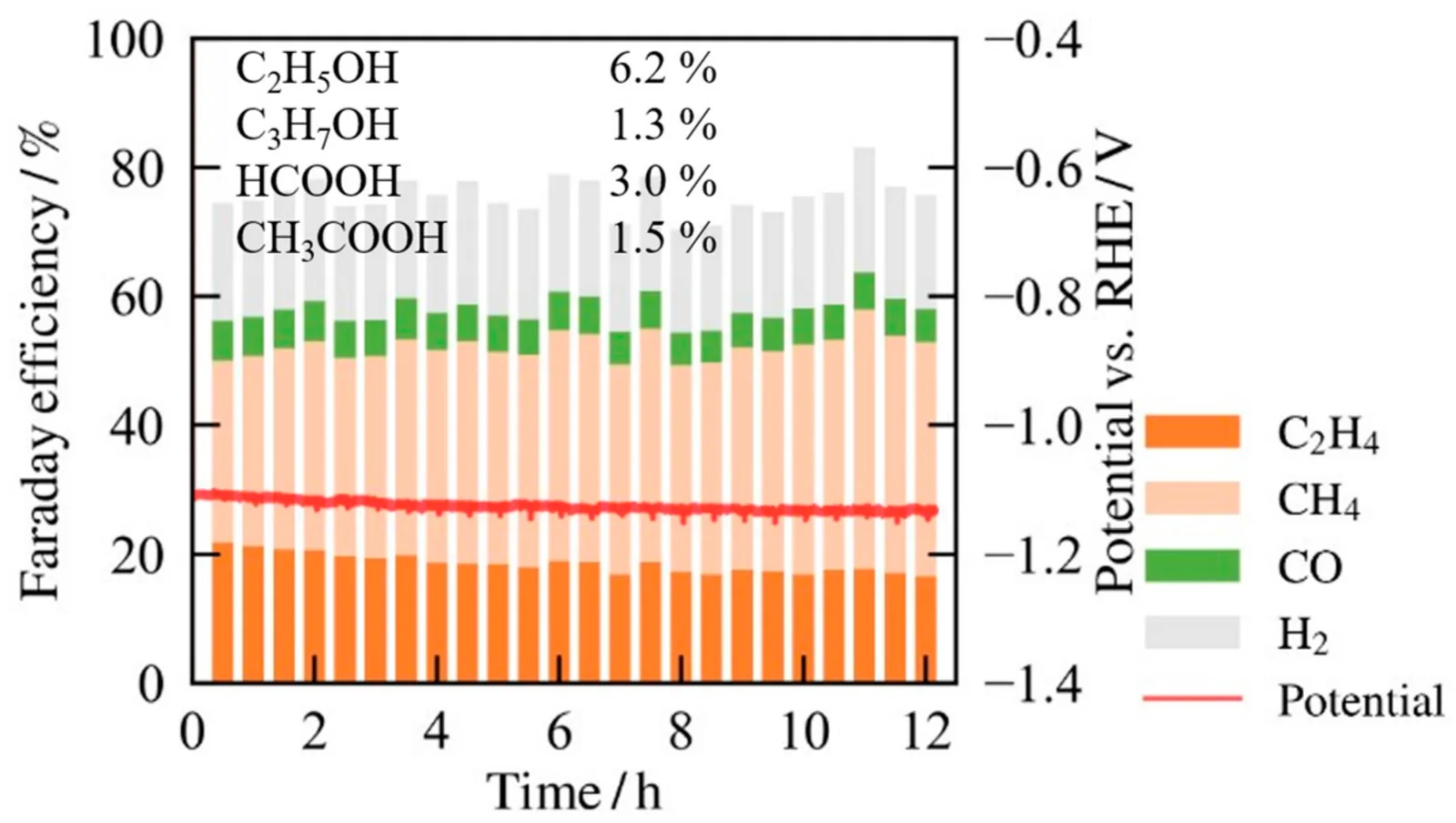
Faraday efficiencies of gaseous products and electrode potential during 12 h of CO_2_ reduction on the Ag-spray-coated Cu electrode at a constant current density of −5 mA/cm^2^ in CO_2_-saturated 0.1 M KHCO_3_. The red line represents the electrode potential (vs. RHE) corrected for ohmic resistance. Numerical values within the plot indicate the Faraday efficiencies of liquid products (C_2_H_5_OH, C_3_H_7_OH, HCOOH, and CH_3_COOH) analyzed after the 12 h reaction.

Figure 4 shows the products of CO_2_ reduction using Ag-spray-coated Cu electrodes and the potential during electrolysis. Unlike the bare Cu electrode, the CO_2_ reduction reaction is active immediately after the reaction starts, with particularly high CH_4_ production. Throughout the 12 h evaluation, the Faradaic efficiency for CH_4_ remains high and stable at approximately 35%. Similarly, the Faradaic efficiency for C_2_H_4_ is maintained at around 18% to 20%. The production of H_2_ is significantly suppressed compared to the bare Cu electrode, staying within a range of 15% to 20% from the beginning of the reaction.

The liquid products analyzed after 12 h show Faradaic efficiencies of 6.2% for C_2_H_5_OH, 1.3% for C_3_H_7_OH, 3.0% for HCOOH, and 1.5% for CH_3_COOH. Reflecting the steady-state operation, the 12 h average FEs for H_2_, CH_4_, and C_2_H_4_ were tightly maintained at approximately 17.5%, 35.0%, and 19.0%, corresponding to average partial current densities of −0.88, −1.75, and −0.95 mA/cm^2^ respectively. The electrode potential is remarkably stable, remaining at approximately −1.12 V vs. RHE. This potential constancy indicates that the Ag-spray-coating effectively bypasses the initial prolonged induction period seen on bare Cu, providing immediate catalytic activation and establishing a robust bimetallic steady-state reaction mode from the onset of electrolysis.

Additionally, CO production is comparable to that of the non-spray-coated Cu electrode at 12 h, as shown in Figure 1. Previous studies have confirmed that when CO_2_ reduction is performed using an Ag electrode, CO is primarily produced as the product. This suggests that the CO generated on the Ag surface is not directly released as a CO product; instead, the CO moves from the Ag surface to the Cu electrode, where further reactions occur, leading to the reduction of CO to CH_4_ and C_2_H_4_.

**Figure 5:**
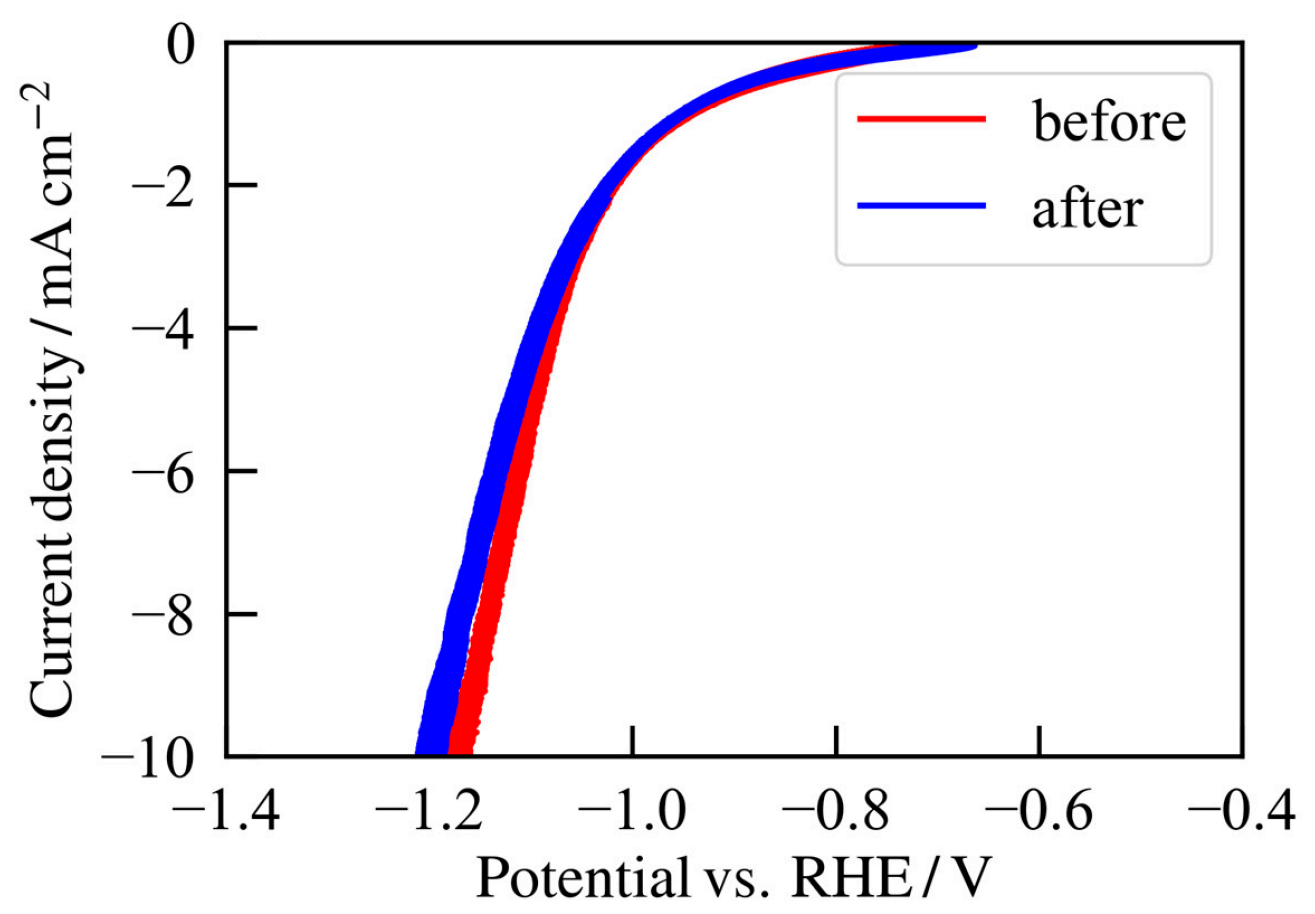
I-V characteristics of the Ag-spray-coated Cu electrode recorded before (red line) and after (blue line) 12 h of electrolysis in CO_2_-saturated 0.1 M KHCO_3_. All potentials were corrected for ohmic resistance (I-R drop) based on EIS measurements. The overlapping curves indicate the high electrochemical stability of the catalyst surface during the reaction.

Figure 5 shows the I-V characteristics of the Ag-spray-coated Cu electrode before and after electrolysis. The current-voltage curves obtained before and after the 12 h electrolysis are almost identical, with the potential required to reach a current density of −10 mA/cm^2^ staying at approximately −1.17 V vs. RHE. This stability in the I-V characteristics suggests that the catalytic activity and the surface state of the electrode remain highly stable throughout the process. This result is consistent with the fact that there was almost no fluctuation in electrode potential during the constant current operation shown in Figure 4.

It is worth noting that the Ag-spray-coated Cu electrode requires a more negative potential to drive the current compared to the initial bare Cu substrate. This behavior reflects a major shift in the dominant reaction pathway; while the rapid current rise on bare Cu is contributed by the kinetically facile hydrogen evolution reaction (HER), the Ag-modified interface selectively drives the complex electrochemical CO_2_ reduction reaction (eCO_2_RR). Because the eCO_2_RR pathways toward hydrocarbons demand intricate multi-electron transfer steps, they inherently require a higher activation overpotential than the simple 2-electron HER, representing an acceptable thermodynamic trade-off for high-value chemical synthesis.

### 2.3. Characterization of Surface Oxidation States via AP-HAXPES

**Figure 6:**
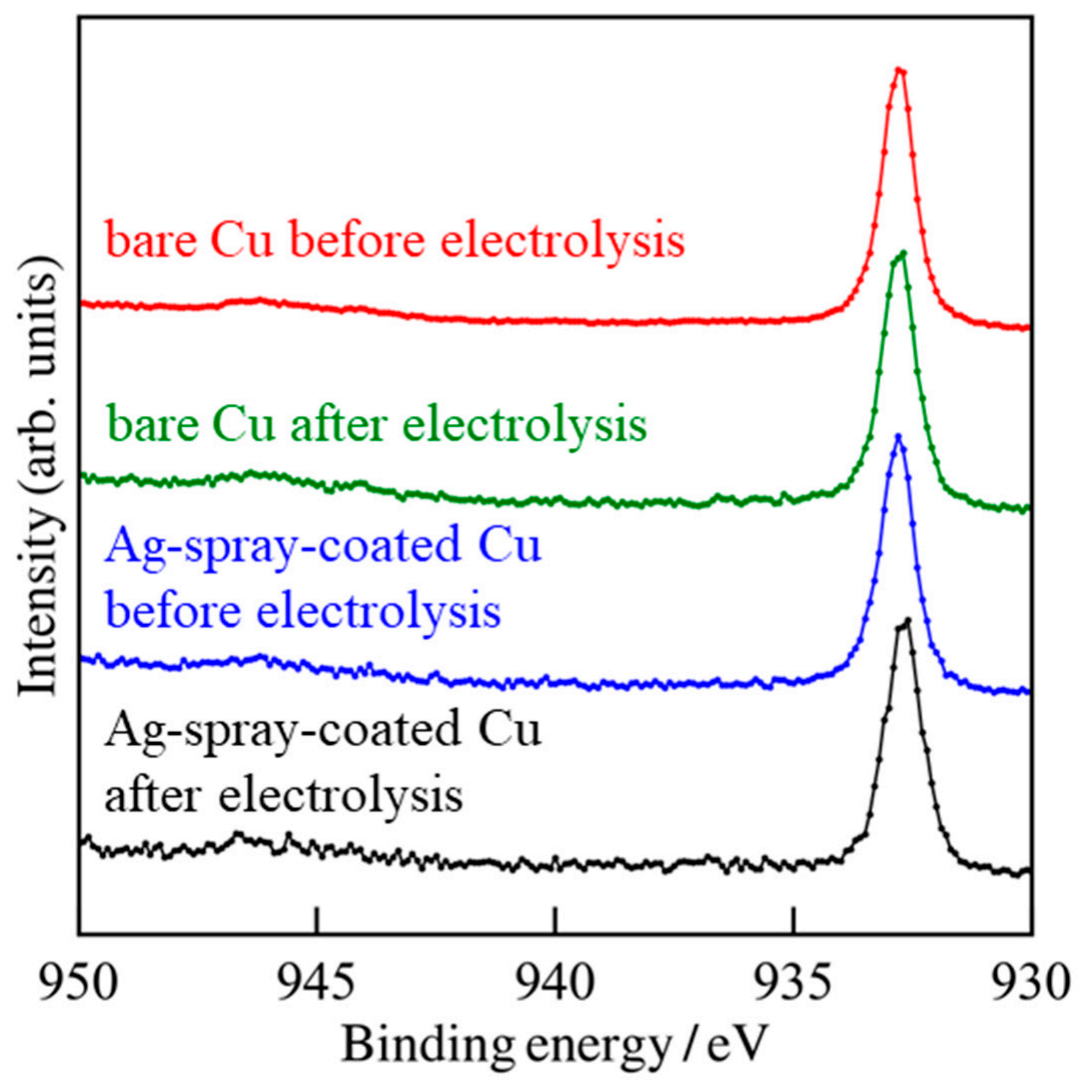
Cu 2p3/2 spectra of the bare Cu and Ag-spray-coated Cu electrodes obtained by AP-HAXPES before and after electrolysis. The primary peaks are located at 932.8 eV for all experimental conditions.

Figure 6 presents the Cu 2p3/2 spectra for the bare Cu and Ag-spray-coated Cu electrodes before and after the 12 h electrolysis. In all four conditions, the primary peak is located at 932.8 eV. Because the Cu 2p3/2 peak positions for metallic Cu and Cu_2_O are located extremely close to each other (~932.4–932.8 eV) and are difficult to resolve independently, the O 1s peak regions were evaluated to accurately distinguish these surface oxidation states. Notably, a subtle shift toward the lower binding energy region was observed exclusively for the Ag-spray-coated Cu electrode after electrolysis, reflecting the unique presence of the Cu_2_O phase under the operational conditions.

**Figure 7:**
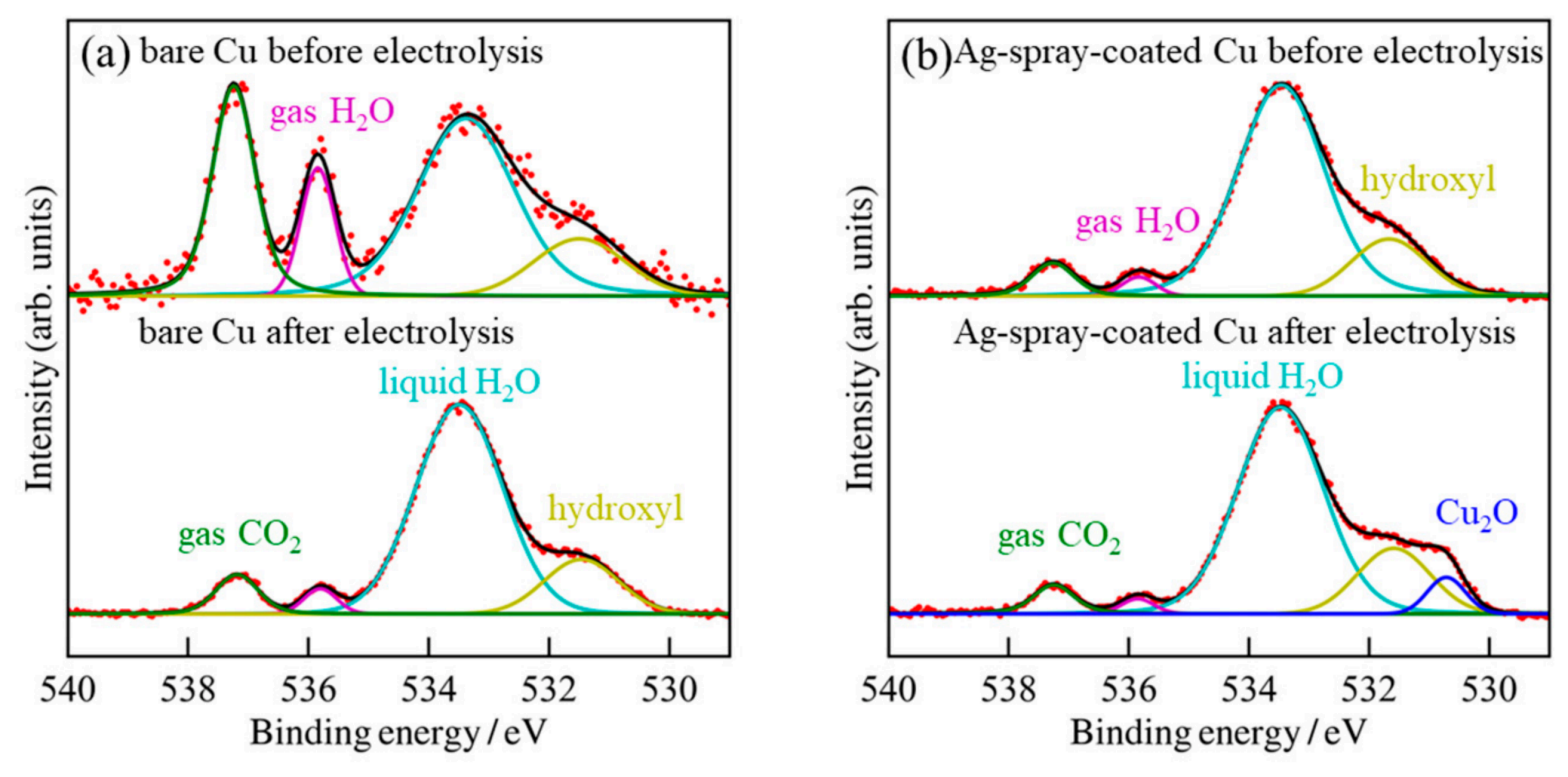
AP-HAXPES spectra of the bare Cu and Ag-spray-coated Cu electrodes before and after 12 h of electrolysis. (**a**) O 1s spectra for the bare Cu electrode. (**b**) O 1s spectra for the Ag-spray-coated Cu electrode. The measurements were performed under an ambient pressure of 4400 Pa in a H_2_O and CO_2_ atmosphere.

Figure 7a,b show the O 1s spectra for the bare Cu and Ag-spray-coated Cu electrodes, respectively. The peak located at approximately 530.7 eV corresponds to the lattice oxygen within the Cu_2_O layer. The peaks between 532.5 eV and 533.5 eV are attributed to H_2_O from the 0.01 M KHCO_3_ electrolyte. The peak near 537 eV is assigned to gas-phase CO_2_, and the peak near 536 eV corresponds to gas-phase H_2_O. The peak at 531.6 eV is considered to originate from surface-adsorbed OH. Notably, the peak derived from Cu_2_O was confirmed only for the Ag-spray-coated Cu electrode after electrolysis.

A strict numerical peak area quantification or deconvolution of the Cu^0^/Cu^+^ ratios was intentionally omitted because the dynamically adsorbed water layer on the electrode surface under the humidified AP-HAXPES environment fluctuates the photoelectron attenuation and effective probing depth. Thus, the analysis focused on the definitive qualitative presence of the lattice oxygen peak at 530.7 eV as the most reliable, artifact-free evidence. It should also be noted that the Ag 3d spectra fell below the reliable detection limit due to the deep volumetric probing depth of hard X-rays relative to the discrete nanoparticle layer and signal attenuation by the electrolyte film. Nevertheless, the dominance of the intermediate spillover mechanism is chemically supported by the immediate surge in deeply reduced hydrocarbons rather than CO release from the Ag sites.

## 3. Discussion

Crucially, this systematic electrochemical evaluation allows for a rigorous, unconfounded comparison of product selectivity under equivalent thermodynamic potentials. As shown in Section 2.2, the Ag-spray-coated Cu electrode operated at −5 mA/cm^2^ exhibits a highly stable operational potential of approximately −1.12 V vs. RHE. Meanwhile, the bare Cu electrode operated at −15 mA/cm^2^ stabilizes at −1.15 V vs. RHE (Section 2.1), providing a near-identical thermodynamic operational window. Despite operating in the exact same potential range (approximately −1.12 to −1.15 V vs. RHE), their catalytic selectivity profiles exhibit a stark contrast. On the bare Cu electrode, the hydrogen evolution reaction (HER) remains overwhelmingly dominant, yielding a high H_2_ FE of 51% with only 27% for CH_4_ and 4.7% for C_2_H_4_. In sharp contrast, the Ag-spray-coated Cu electrode successfully suppresses the competing HER to approximately 17.5% while achieving superior selectivities for deeply reduced hydrocarbons (CH_4_: ~35%, C_2_H_4_: ~19%).

This direct comparison under equivalent potentials definitively rules out the applied potential as the primary origin of the enhanced hydrocarbon selectivity. It demonstrates that driving bare Cu to more negative potentials simply accelerates the kinetically rapid HER under these stationary aqueous conditions. Conversely, the presence of the Ag-spray-coating dynamically alters the interfacial reaction pathway. This synergistic improvement is highly consistent with the intermediate CO spillover mechanism, where CO intermediates generated on the active Ag sites migrate to the adjacent Cu sites for further deep reduction, bypass the initial induction period, and effectively prevent the domination of HER. It is important to acknowledge, however, that comparing selectivity across different current densities (−5 mA/cm^2^ for Ag/Cu vs. −15 mA/cm^2^ for bare Cu) involves distinct micro-interfacial reaction environments, including variations in local surface pH, CO_2_ concentration gradients, and mass-transport dynamics. On bare Cu operated at a higher current density of −15 mA/cm^2^, the increased current density elevates the local interfacial pH—a condition intrinsically unfavorable for HER. Despite this unfavorable local pH environment, hydrogen evolution remains overwhelmingly dominant (H_2_ FE: 51.41%), reflecting localized CO_2_ mass-transport limitations and the lack of intermediate CO enrichment on the bare Cu surface.

In our evaluation, the system is assessed under two complementary frameworks: at the same total current density (−5 mA/cm^2^, Figure 1 and Figure 4) and at matched operational potentials (~−1.12 to −1.15 V vs. RHE). The stark divergence in product distributions observed across both comparison metrics consistently demonstrates that Ag nanoparticle modification—via localized CO enrichment and intermediate spillover—plays a dominant role in steering the reaction pathway away from HER and toward selective hydrocarbon production.

For the bare Cu electrode, although the reduction products shifted from H_2_ to hydrocarbons during the first two hours, the HAXPES results suggest the copper surface remains as metallic Cu under these conditions. Although these quasi-in situ AP-HAXPES configurations do not perfectly mirror the bulk electrolysis environment, the clear relative trends demonstrate that the Ag-spray-coated Cu interface possesses an intrinsic chemical capability to sustain the oxidized copper species (Cu_2_O) under cathodic polarization, whereas the bare Cu surface is fully reduced to metallic Cu. This observed shift toward CO_2_ reduction is likely caused by the gradual coverage of the electrode surface with CO, which eventually inhibits the hydrogen evolution reaction [22,23,24]. 

**Figure 8:**
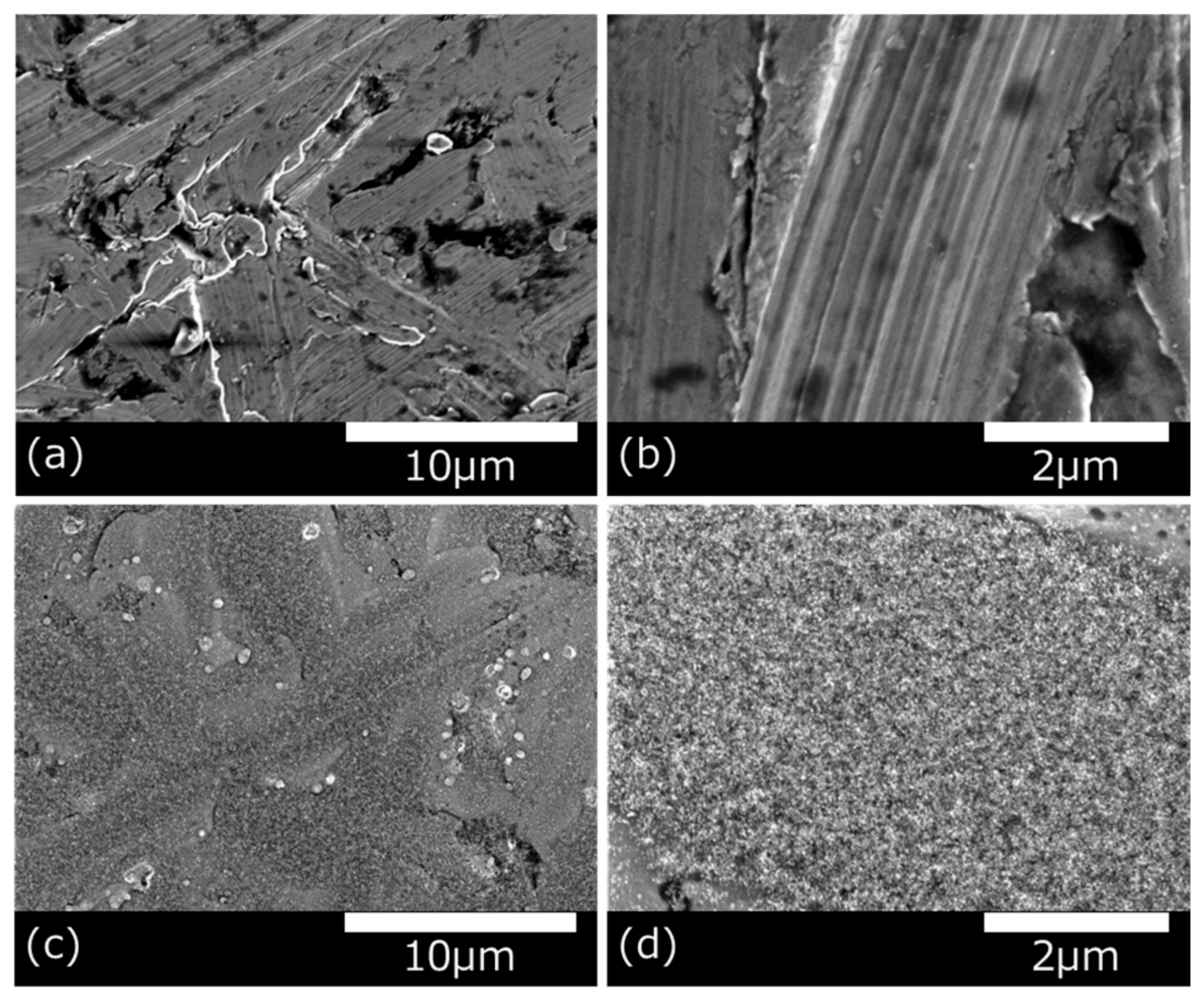
SEM images of the electrodes at different magnifications. (**a**,**b**) Bare Cu electrodes. Surface scratches from machining are observed. (**c**,**d**) Ag-spray-coated Cu electrodes. Silver particles are seen deposited on the surfaces.

Figure 8 shows the SEM images of both electrodes at different magnifications. However, since SEM observations revealed no significant morphological changes beyond minor machining scratches, the specific surface alterations or changes in chemical bonding connecting the modification of the CO adsorption state remain unclear based on the current data. Preliminary SEM-EDS analyses confirmed a broad distribution of Ag across the Cu substrate; however, owing to the spatial resolution constraints and X-ray excitation volume of conventional EDS, local correlation between Ag signals and specific nanoscale morphological features remained unresolved. To fully map the fine interfacial redistribution or thermal interdiffusion of Ag species at the nanoscale, further high-resolution techniques, such as high-resolution TEM or STEM-EDS, will be required in future work.

In the Ag-spray-coated Cu electrode, the copper surface was found to exist as Cu_2_O during electrolysis. Although the complete reduction of copper oxides to metallic Cu is thermodynamically expected under intensive cathodic conditions, and the precise fundamental mechanism underlying this stabilization remains not fully understood at this stage, the clear persistence of the Cu_2_O phase is an undeniable experimental fact captured by our AP-HAXPES measurements. The immediate and stable production of CH_4_ and C_2_H_4_ is considered to be correlated with this experimentally confirmed Cu_2_O surface state. Specifically, CO generated on the Ag nanoparticles immediately after the start of electrolysis migrates to the Cu surface. This process inhibits hydrogen generation and facilitates the reduction from CO to CH_4_ and C_2_H_4_ on the Cu surface. The improvement in selectivity is attributed to a spillover mechanism where CO generated on the Ag nanoparticles migrates to adjacent Cu_2_O sites for further reduction to hydrocarbons [14,22,23,24]. This indicates that surface modification with Ag nanoparticles facilitates a more efficient reaction pathway from the start of electrolysis. It should be noted, however, that while the macroscopic product selectivity profiles at matched potentials and the AP-HAXPES surface state evaluations provide strong, consistent support for the CO spillover hypothesis, direct observational proof of intermediate migration remains to be fully established. Based on the present data alone, alternative synergistic mechanisms at the bimetallic interface cannot be entirely ruled out. To definitively track the real-time spatial migration and reaction dynamics of CO intermediates from Ag to Cu sites, further direct operando studies—such as in situ Fourier-transform infrared spectroscopy (FTIR), differential electrochemical mass spectrometry (DEMS), or ^13^C isotopic labeling experiments—will be conducted in future work.

In this context, the relatively high Ag loading of 1000 μg/cm^2^ is considered to play a significant role in influencing the local reaction environment. Rather than acting as a sparse decoration, this loading is thought to establish a continuous, porous nanoparticle layer over the Cu substrate, as evidenced by SEM (Figure 8c,d). This high-density Ag layer is hypothesized to act as an abundant CO generation domain, potentially creating a localized, CO-rich microenvironment at the bimetallic interface. Such localized enrichment of CO intermediates may facilitate their subsequent transfer and spillover to adjacent Cu/Cu_2_O active sites for further reduction into CH_4_ and C_2_H_4_. While such a thick catalytic layer could introduce interfacial mass-transfer limitations, the localized enrichment of CO intermediates is considered a plausible factor contributing to steering the reaction pathway toward selective hydrocarbon production.

These findings suggest that the long-term stability of the Ag-spray-coated Cu electrode is supported by the maintenance of the Cu_2_O surface state and the integrated physical structure of the bimetallic catalyst. While the bare Cu electrode eventually achieves a product distribution similar to the Ag-modified electrode, the Ag-spray-coating effectively bypasses the initial induction period, providing superior catalytic consistency. It should be emphasized that while the 800 °C annealing was necessary for flat laboratory bulk foils, this thermal budget can be entirely bypassed in practical industrial upscaling. In commercial zero-gap cell configurations utilizing gas diffusion layers (e.g., carbon paper), the catalyst layer is mechanically clamped tightly against the membrane, naturally suppressing catalyst peeling. As documented in the previous literature [25], non-templated Ag-spray-coated Cu configurations can exhibit altered and enhanced catalytic properties even without such thermal treatments, preserving the low-cost and facile advantages of the room-temperature spray-deposition approach for scalable applications. 

## 4. Materials and Methods

### 4.1. Sample Preparation

**Figure 9:**
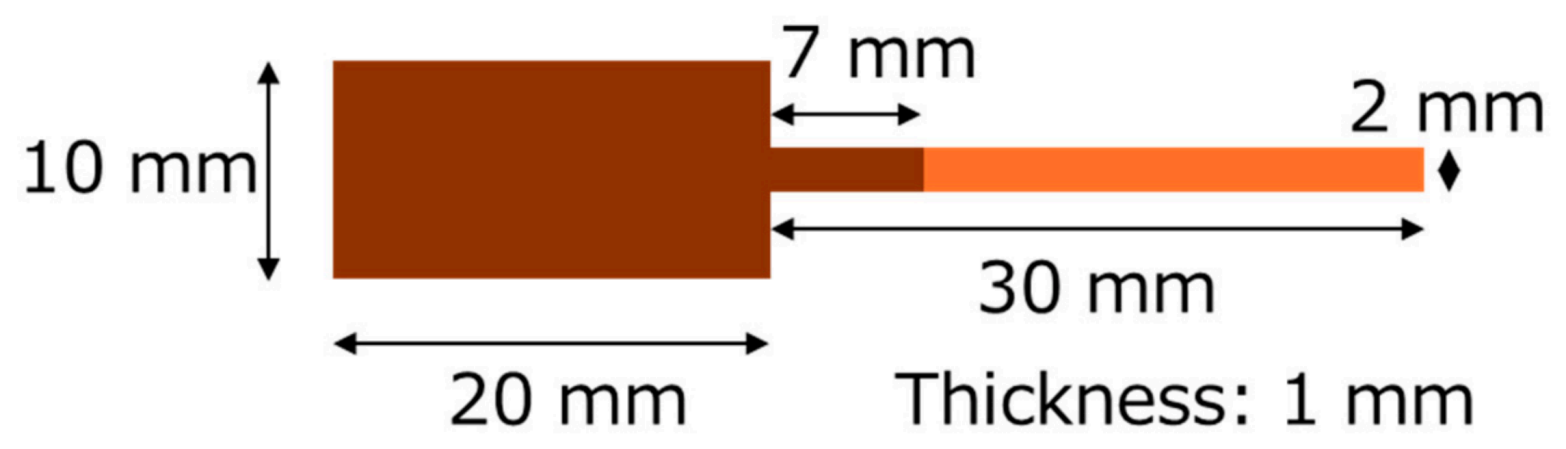
Schematic and dimensions of the Cu working electrode. The darker shaded area corresponds to the portion immersed in the electrolyte. The total geometric surface area exposed to the solution is 5 cm^2^, calculated from the combined area of both sides and the edges. The electrode has a constant thickness of 1 mm, and the narrower section is used for electrical connection.

In this study, two types of electrodes were prepared and evaluated: Ag-spray-coated Cu electrodes and bare Cu electrodes as a reference. The base Cu electrodes (Nilaco, Tokyo, Japan, CU-113511) were fabricated into the geometry shown in Figure 9.

The Ag-modified electrodes were fabricated by spray-coating an Ag nanoparticle ink onto the Cu substrate. The ink was prepared by diluting 1 mL of an Ag nanoparticle dispersion (US Research Nanomaterials, Houston, TX, USA; 10,000 ppm, 15 nm particle size) in 50 mL of ethanol. The spray-coating process was adjusted to reach a target Ag mass loading of 1000 μg/cm^2^ using an automated fine-mist nozzle with a fixed spray distance of 15 cm and a carrier gas pressure of 0.3 MPa. To ensure robust mechanical adhesion, the electrodes were annealed at 800 °C for 10 min in a hydrogen (H_2_) atmosphere. In preliminary trials without this thermal treatment, the spray-coated Ag nanoparticles were observed to detach from the Cu substrate upon electrolyte immersion and CO_2_ bubbling. The 800 °C annealing completely prevented this detachment, allowing the Ag layer to remain firmly attached even during vigorous, long-term gas-evolving electrolysis. This dramatic enhancement in adhesion strongly suggests that the thermal process induces interfacial atomic diffusion or localized bimetallic bonding between Ag and Cu. Although direct structural identification of the exact interfacial alloy phase remains to be confirmed, this annealing step is functionally essential for establishing a mechanically stable bimetallic interface for evaluation.

The bare Cu electrode exhibits distinct linear scratches on its surface, resulting from the mechanical machining process (Figure 8a,b). In contrast, the Ag-spray-coated Cu electrode displays a surface successfully covered with Ag nanoparticles (Figure 8c,d). At higher magnification, these nanoparticles appear to form a porous and integrated structure on the Cu substrate.

Prior to the electrochemical measurements, a specific cleaning protocol was implemented at room temperature to ensure the complete removal of surface oxides and contaminants. Each electrode was first immersed in 1 M sulfuric acid for 30 s. Subsequently, the electrodes were thoroughly rinsed with ultrapure water, followed by a final rinse with ethanol. The electrodes were then dried before being immediately integrated into the electrochemical cell. 

### 4.2. Electrochemical Measurements

**Figure 10:**
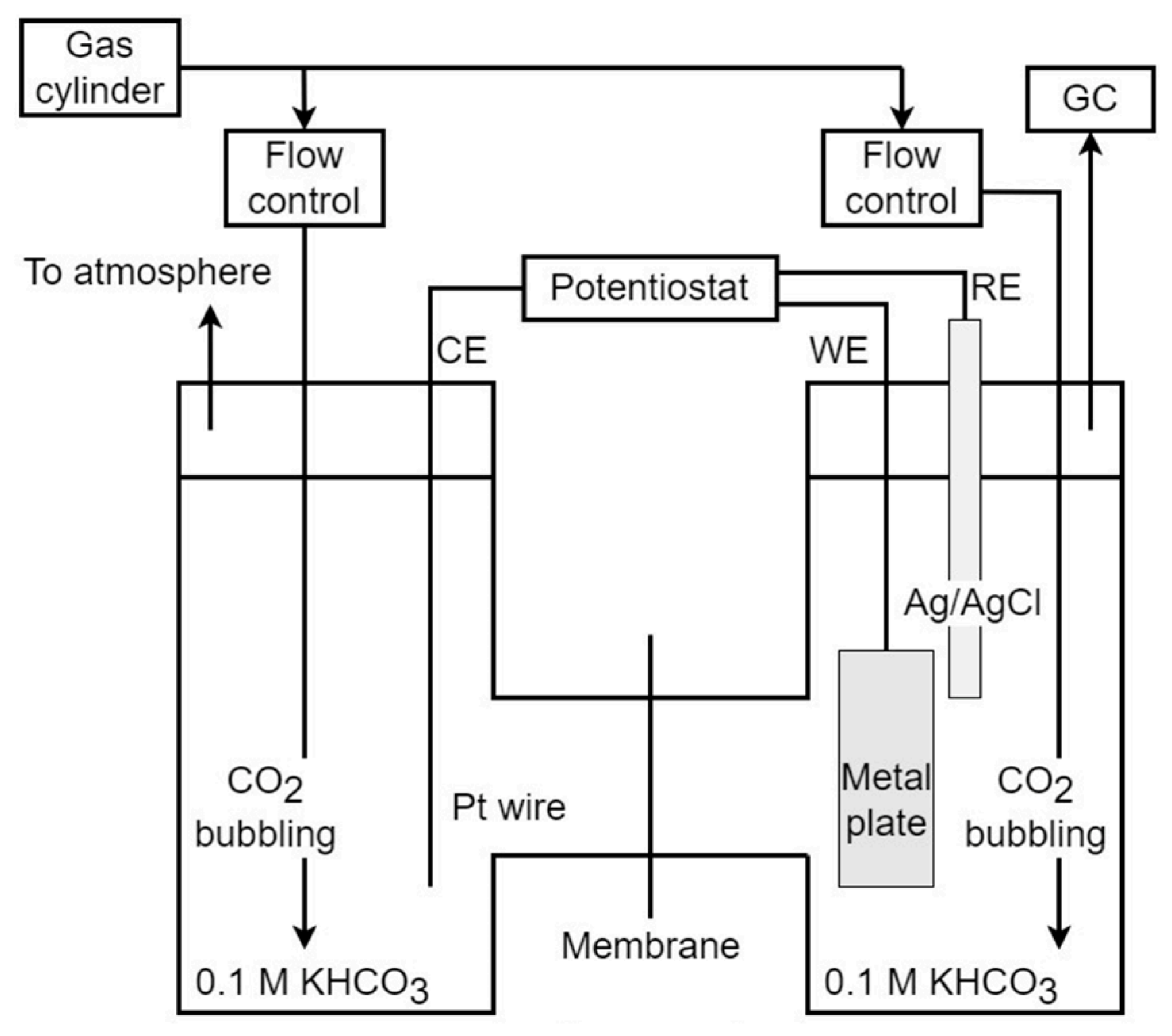
Schematic illustration of the experimental setup for electrochemical CO_2_ reduction. The H-type electrolytic cell consists of two compartments separated by an ion-exchange membrane. 0.1 M KHCO_3_ is used as the electrolyte with continuous CO_2_ bubbling. A three-electrode system is employed, featuring a metal plate working electrode (WE), a Pt wire counter electrode (CE), and an Ag/AgCl reference electrode (RE). The gas phase is connected to a gas chromatograph (GC) for product analysis.

Figure 10 illustrates the schematic of the electrochemical measurement setup. An H-type cell consisting of two compartments separated by an anion exchange membrane (AEM; AGC Selemion ASVN, Chiba, Japan) was employed. Each compartment was filled with 37 mL of 0.1 M KHCO_3_ electrolyte, which was pre-saturated with CO_2_. The working electrode (WE) and an Ag/AgCl reference electrode (RE; BAS Inc. RE-1B, Tokyo, Japan) were placed in the cathodic compartment, while a Pt wire (Nilaco PT-351384, Tokyo, Japan) served as the counter electrode (CE) in the anodic compartment. The potential of the Ag/AgCl reference electrode was 0.61 V vs. RHE in the CO_2_-saturated 0.1 M KHCO_3_ electrolyte.

An AEM was specifically selected to maintain a stable electrolyte concentration during prolonged electrolysis. Unlike cation exchange membranes, which allow potassium ion migration from the anode to the cathode (leading to undesirable concentration gradients based on our pre-evaluations), the AEM effectively suppresses this migration. The electrochemical potential and current were controlled and monitored using a potentiostat (Bio-Logic Science Instruments SP-300, Seyssinet-Pariset, France). All electrode potentials reported in this study were corrected for ohmic resistance (I-R drop) determined by electrochemical impedance spectroscopy (EIS).

CO_2_ was continuously bubbled into both compartments at a constant flow rate. The exhaust gas from the cathodic side was directly routed to a gas chromatograph (GC; Agilent 8890, Santa Clara, CA, USA) for real-time analysis of gaseous products, with the CO_2_ flow acting as the carrier gas. Gaseous products were sampled and analyzed every 30 min using a Thermal Conductivity Detector (TCD). The Faradaic efficiency (FE) for each product was calculated based on these measured concentrations.

Liquid products were analyzed post-electrolysis to determine the average FE over the reaction period. Alcohols were quantified by direct injection into the GC equipped with a Flame Ionization Detector (FID). Organic acids were first esterified to enhance detection sensitivity before being analyzed via GC-FID. The details are described in the Supplementary Materials (Sections S1 and S2). For stability testing, constant-current electrolysis was performed at a current density of −5 mA/cm^2^ for 12 h. Current-voltage (I-V) characteristics were recorded both before and after the 12 h electrolysis period by sweeping the current density from 0 to −10 mA/cm^2^. The 12 h bulk electrolysis data presented in this study were collected from representative continuous operational runs. To ensure the physical and catalytic reproducibility of the Ag nanoparticle spray-coating method, independent short-term (3 h) electrolysis evaluations were performed using separately fabricated electrodes prepared under the identical protocol (Figure S1, Supporting Information). These tests confirmed highly consistent product selectivities and Faradaic efficiencies for all major gaseous products (CH_4_, C_2_H_4_, CO, and H_2_), validating the robustness and reproducibility of the catalyst preparation.

### 4.3. AP-HAXPES Characterization

To investigate the oxidation states of the electrode surfaces under reaction conditions, ambient pressure hard X-ray photoelectron spectroscopy (AP-HAXPES) was performed at beamline BL46XU of SPring-8 (Sayo, Japan). The incident X-ray energy was tuned to 7940 eV using a Si(111) double-crystal monochromator and a downstream Si(311) double-channel cut monochromator. Photoelectrons were collected using a Scienta Omicron R4000-Hipp2 (Taunusstein, Germany) differential pumping analyzer, operated at a pass energy of 200 eV with a 0.5 mm slit and a 70 μm diameter circular aperture. The binding energy scale and resolution were calibrated using the Au 4f spectrum as a reference.

The measurements were conducted in an environmental chamber maintained at 4400 Pa under an H_2_O and CO_2_ atmosphere. A beaker-type electrochemical cell was integrated into the chamber, containing a 0.01 M KHCO_3_ aqueous solution. The electrolyte concentration was intentionally decreased to 0.01 M (compared to the 0.1 M used in bulk electrolysis) as an experimentally unavoidable measure to prevent localized salt crystallization at the meniscus interface under reduced pressure and intense X-ray irradiation. Furthermore, due to strict spatial constraints inside the specialized beamline cell, a reference electrode could not be integrated, and the potential was controlled against a Pt wire counter electrode (−2.4 V vs. CE) for 10 min. Subsequently, the electrode was partially extracted from the electrolyte while maintaining the applied potential to ensure the stability of the surface species during the HAXPES data acquisition.

In this study, four distinct conditions were characterized: (i) bare Cu before electrolysis, (ii) bare Cu after the formal 12 h bulk electrolysis, (iii) Ag-spray-coated Cu before electrolysis, and (iv) Ag-spray-coated Cu after the formal 12 h bulk electrolysis. For the post-electrolysis samples, a brief 10 min polarization at −2.4 V vs. CE was applied inside the HAXPES cell prior to data acquisition strictly to electrochemically remove superficial native oxides formed during sample transfer through ambient air. The analysis focused on the Cu 2p3/2 and O 1s spectra. For the Cu 2p3/2 spectra, the primary peak for metallic Cu and Cu_2_O is expected at approximately 932.4 eV, with Cu_2_O identified by its characteristic shake-up features at 938.0 and 946.0 eV [26]. For CuO, the binding energy shifts to 933.6 eV, accompanied by a distinct satellite peak at 942.0 eV [26]. In the O 1s spectra, the following peaks were analyzed: lattice oxygen in the Cu_2_O layer at 530.7 eV, surface adsorbed OH at 531.6 eV, H_2_O from the electrolyte between 532.5 eV and 533.5 eV, gas phase H_2_O at 536 eV, and gas phase CO_2_ at 537 eV [26,27].

## 5. Conclusions

In this study, we evaluated the electrochemical CO_2_ reduction performance and surface stability of bare Cu and Ag-spray-coated Cu electrodes. For the bare Cu electrode, the reaction initially produced nearly 100% H_2_, followed by a gradual shift toward hydrocarbon production after approximately two hours. This transition was accompanied by a significant negative shift in both the electrode potential and the current-voltage characteristics, indicating that the bare Cu surface requires increasing energy as the CO_2_ reduction reaction proceeds.

In contrast, the Ag-spray-coated Cu electrode exhibited immediate and stable activity for hydrocarbon production. Throughout the 12 h evaluation, the Faradaic efficiencies for CH_4_ and C_2_H_4_ were maintained at approximately 35% and 20%, respectively, while H_2_ generation was effectively suppressed. The electrode potential and I-V characteristics remained remarkably stable, suggesting that the Ag-spray-coated Cu surface is more resistant to time-dependent changes during electrolysis.

AP-HAXPES analysis revealed that while the bare Cu surface remains metallic, the copper surface of the Ag-modified electrode exists as Cu_2_O during the reaction. The enhanced performance and stability are attributed to a spillover mechanism, where CO generated on the Ag nanoparticles migrates to adjacent Cu. This process inhibits hydrogen generation and facilitates the efficient reduction from CO_2_ to CH_4_ and C_2_H_4_ from the start of electrolysis. These findings demonstrate that surface modification using the facile Ag nanoparticle spray-coating method is a highly effective approach for achieving both high product selectivity and long-term stability in electrochemical CO_2_ reduction.

## Data Availability

The raw data supporting the conclusions of this article will be made available by the authors on request.

## Supplementary Materials

The following supporting information can be downloaded at: https://www.mdpi.com/article/10.3390/molecules31162803/s1, Section S1: Analysis of Organic Acids (Formic and Acetic Acids); Section S2: Analysis of Alcohols; Section S3: Short-Term (3 h) Bulk Electrolysis Evaluation of Ag-Spray-Coated Cu Electrode. Figure S1: Faradaic efficiencies of gaseous products (CH_4_, C_2_H_4_, CO, and H_2_) and cell voltage during 3 h of continuous CO_2_ reduction using the Ag-spray-coated Cu electrode at a constant current density of −10 mA/cm^2^.

## References

[1] Abernethy S., Jackson R.B. (2022). Global temperature goals should determine the time horizons for greenhouse gas emission metrics. Environ. Res. Lett..

[2] FCCC/CP/2015/L.9/Rev.1. https://unfccc.int/resource/docs/2015/cop21/eng/l09r01.pdf.

[3] IPCC_AR6_WGI_SPM. https://www.ipcc.ch/report/ar6/wg1/downloads/report/IPCC_AR6_WGI_SPM.pdf.

[4] Peres C.B., Resende P.M.R., Nunes L.J.R., de Morais C. (2022). Advances in Carbon Capture and Use (CCU) Technologies: A Comprehensive Review and CO_2_ Mitigation Potential Analysis. Clean Technol..

[5] Hori Y., Wakebe H., Tsukamoto T., Koga O. (1994). Electrocatalytic process of CO selectivity in electrochemical reduction of CO_2_ at metal electrodes in aqueous media. Electrochim. Acta.

[6] Hori Y., Vayenas C., White R., Gamboa-Aldeco M. (2008). Electrochemical CO_2_ Reduction on Metal Electrodes. Modern Aspects of Electrochemistry.

[7] Buckley A.K., Cheng T., Oh M.H., Su G.M., Garrison J., Utan S.W., Zhu C., Toste F.D., Goddard W.A., Toma F.M. (2021). Approaching 100% Selectivity at Low Potential on Ag for Electrochemical CO_2_ Reduction to CO Using a Surface Additive. ACS Catal..

[8] Hori Y., Takahashi R., Yoshinami Y., Murata A. (1997). Electrochemical Reduction of CO at a Copper Electrode. J. Phys. Chem. B.

[9] Hori Y., Murata A., Takahashi R., Suzuki S. (1987). Electroreduction of carbon monoxide to methane and ethylene at a copper electrode in aqueous solutions at ambient temperature and pressure. J. Am. Chem. Soc..

[10] Mistry H., Reske R., Strasser P., Cuenya B.R. (2017). Size-dependent reactivity of gold-copper bimetallic nanoparticles during CO_2_ electroreduction. Catal. Today.

[11] Higgins D., Landers A.T., Ji Y., Nitopi S., Morales-Guio C.G., Wang L., Chan K., Hahn C., Jaramillo T.F. (2018). Guiding Electrochemical Carbon Dioxide Reduction toward Carbonyls Using Copper Silver Thin Films with Interphase Miscibility. ACS Energy Lett..

[12] Clark E.L., Hahn C., Jaramillo T.F., Bell A.T. (2017). Electrochemical CO_2_ Reduction over Compressively Strained CuAg Surface Alloys with Enhanced Multi-Carbon Oxygenate Selectivity. J. Am. Chem. Soc..

[13] Shen S., Peng X., Song L., Qiu Y., Li C., Zhuo L., He J., Ren J., Liu X., Luo J. (2019). AuCu Alloy Nanoparticle Embedded Cu Submicrocone Arrays for Selective Conversion of CO_2_ to Ethanol. Small.

[14] Liu Y., Qiu H., Li J., Guo L., Ager J.W. (2021). Tandem Electrocatalytic CO_2_ Reduction with Efficient Intermediate Conversion over Pyramid-Textured Cu–Ag Catalysts. ACS Appl. Mater. Interfaces.

[15] Cai J., Zhao Q., Hsu W.Y., Choi C., Liu Y., Martirez J.M.P., Chen C., Huang J., Carter E.A., Huang Y. (2023). Highly Selective Electrochemical Reduction of CO_2_ into Methane on Nanotwinned Cu. J. Am. Chem. Soc..

[16] Jeon H.S., Kunze S., Scholten F., Cuenya B.R. (2018). Prism-Shaped Cu Nanocatalysts for Electrochemical CO_2_ Reduction to Ethylene. ACS Catal..

[17] Dong H., Zhang Z., Zhou D., Huang S., Lin Z., Zhang X., Zhang Z., Lei J., Liu N. (2026). Progress of photothermal/thermal catalytic CO_2_ hydrogenation by metal-modified CeO_2_. Green Chem..

[18] Zhang Z., Huang W., Dong H., Cui H., Zhou D., Lin Z., Hu B., Zhang X., Huang S., Tang L. (2026). Self-Tuned Ligand-to-Metal–Metal Charge Transfer in Node-Engineered Al-Doped NH_2_-UiO-66 Boosts CO_2_ Photoreduction Activity. Adv. Funct. Mater..

[19] Cui H., Li Z., Huang S., Zhang T., Zhang X., Zhang Z., Lei J., Liu N. (2026). Cu/TiO_2_ Derived from Cu-Doped MIL-125 for Enhanced Photocatalytic CO_2_-to-CH_4_ Conversion. Molecules.

[20] Ke X., Xu W., Liu C., Wang Y., Huang X., Xiao R., Xu X., Li T., Shao Z. (2025). Innovative electrode designs for low-temperature electrochemical CO_2_ reduction with ampere-level performance. Energy Environ. Sci..

[21] Liu M., Zhang C., Ying Y., Zhao Y., Zhao Z., Jia Y., Chen Y., Shi J., Li Y. (2025). Optimization strategies for enhancing the stability of Cu-based catalysts. Mater. Rep. Energy.

[22] Osiewacz J., Löffelholz M., Weseler L., Turek T. (2023). Selective C–C Coupling in Carbon Dioxide Electroreduction via Efficient Spillover of Intermediates As Supported by Operando Raman Spectroscopy. Electrochim. Acta.

[23] Chen X., Granda-Marulanda L.P., McCrum I.T., Koper M.T.M. (2022). Nanoscale Cu–Ag Heterostructures for CO_2_ Reduction to C_2+_ Products. Nat. Commun..

[24] Akhade S.A., Luo W., Nie X., Bernstein N.J., Asthagiri A., Janik M.J. (2014). Cu_2_O-Ag Tandem Catalysts for Selective Electrochemical Reduction of CO_2_ to C_2_ Products. Phys. Chem. Chem. Phys..

[25] Koike K., Nara M., Fukushima M., Bae H., Ha H., Fujii K. (2022). Effects of Ag Nanoparticle Coated Metal Electrodes on Electrochemical CO_2_ Reduction in Aqueous KHCO_3_. Electrochemistry.

[26] Jiang P., Prendergast D., Borondics F., Porsgaard S., Giovanetti L., Pach E., Newberg J., Bluhm H., Besenbacher F., Salmeron M. (2013). Experimental and theoretical investigation of the electronic structure of Cu_2_O and CuO thin films on Cu(110) using X-ray photoelectron and absorption spectroscopy. J. Chem. Phys..

[27] Koitaya T., Yamamoto S., Shiozawa Y., Yoshikura Y., Hasegawa M., Tang J., Takeuchi K., Mukai K., Yoshimoto S., Matsuda I. (2019). CO_2_ Activation and Reaction on Zn-Deposited Cu Surfaces Studied by Ambient-Pressure X-ray Photoelectron Spectroscopy. ACS Catal..

