# Supplementary material for: Effect of Surface Modification of Cu Electrodes by Ag Nanoparticle Spray Coating on the Products and Electrolytic Potential of Electrochemical CO2 Reduction
Source: Molecules. 2026 Aug 12;31(16):2803. doi: 10.3390/molecules31162803 (PMC13515030; doi:10.3390/molecules31162803)
Supplement: Supplementary file 1 [file molecules-31-02803-s001.zip › molecules-4479184-supplementary.pdf]

Supporting Information

Effect of surface modification of Cu electrodes by Ag nanoparticle spray coating on the products and electrolytic potential of electrochemical CO_2 reduction

Kazuki Koike [1,2,*], Takeharu Murakami [2], Kentaro Inoue [1], Takayo Ogawa [2], Katsushi Fujii [2], Satoshi Wada [2] and Atsushi Ogura [1,3]

[1] School of Science and Technology, Meiji University, 1-1-1 Higashi-Mita, Tama-ku, Kawasaki 214-8571, Kanagawa, Japan; meijikenino@gmail.com (K.I.); a_ogura@meiji.ac.jp (A.O.)
[2] RIKEN RAP, 2-1 Hirosawa, Wako 351-0198, Saitama, Japan; takeharu.murakami@riken.jp (T.M.); pogawa@riken.jp (T.O.); katsushi.fujii@riken.jp (K.F.); swada@riken.jp (S.W.)
[3] Meiji Renewable Energy Laboratory, Kawasaki 214-8571, Kanagawa, Japan
* Correspondence: kazuki_koike_yz@meiji.ac.jp

S1. Analysis of Organic Acids (Formic and Acetic Acids)

S2. Analysis of Alcohols

S3. Short-Term (3 h) Bulk Electrolysis Evaluation of Ag-Spray-Coated Cu Electrode

S1. Analysis of Organic Acids (Formic and Acetic Acids)

S1.1. GC Measurement Conditions for Formic and Acidic Acids

Derivatization Procedure Formic acid and acetic acid produced during the reaction exhibit low sensitivity when using a Flame Ionization Detector (FID). To improve detection limits and peak resolution, these organic acids were analyzed after an automated derivatization process. Analysis was performed using an Agilent 8890 gas chromatograph (GC) equipped with an Agilent 7693 autosampler.

For the sample preparation, 500 microliters of the electrolyte was placed into a glass vial with boiling stones. The derivatization was automatically conducted using the pre-treatment function of the autosampler. To ensure high throughput, the system was configured to prepare the next sample while the current one was being analyzed. The automated steps for the derivatization (simplified) are as follows:

1. Addition of 50 microliters pyridine.
2. Agitation at 3500 rpm for 20 seconds.
3. Addition of 50 microliters isobutanol.
4. Agitation at 3500 rpm for 20 seconds (repeated 6 times).
5. Addition of 50 microliters isobutyl chloroformate.
6. Incubation for 4 minutes.
7. Agitation at 3500 rpm for 20 seconds (repeated 2 times).
8. Addition of 125 microliters n-hexane from vial B1 (back turret).
9. Addition of 125 microliters n-hexane from vial B2 (back turret).
10. Addition of 125 microliters n-hexane from vial B3 (back turret).
11. Addition of 125 microliters n-hexane from vial B4 (back turret).
12. Agitation at 3500 rpm for 20 seconds (repeated 2 times).
13. Separation for 1 minute.

Note: Hexane (total 500 microliters) was dispensed from four separate vials (B1 to B4) to prevent volume depletion during continuous automated preparation cycles, as the autosampler followed a fixed sequence for reagent sampling. After the separation, the upper hexane layer was sampled and injected into the GC for analysis.

S1.2. GC Measurement Conditions for Organic Acids

- Detector: FID (Heater: 250 degrees C, Air flow: 400 mL/min, H2 flow: 30 mL/min, Makeup flow (N2): 30 mL/min)
- Column: DB-5MS-UI (P/N 122-5533UI)
- Oven Temperature: Initial 40 degrees C (hold 5 min), then ramped at 10 degrees C/min to 250 degrees C (hold 15 min)
- Inlet: Heater at 250 degrees C, Split ratio 50:1

- Carrier Gas: He at 1.0 mL/min (constant flow mode)
- Injection Volume: 1 microliter

S2. Analysis of Alcohols

S1.1. GC Measurement Conditions for Formic and Acidic Acids

The concentration of alcohol products in the electrolyte was determined by direct injection into a gas chromatograph. An Agilent 7890B GC system equipped with an FID was used for the quantification of liquid products such as ethanol (C_2H_5OH) and propanol (C_3H_7OH).

S2.1. GC Measurement Conditions for Alcohols

- Detector: FID (Heater: 250 degrees C, Air flow: 400 mL/min, H2 flow: 30 mL/min, Makeup flow (N2): 30 mL/min)
- Column: DB-FFAP (P/N 125-3232)
- Oven Temperature: Initial 40 degrees C (hold 1 min), then ramped at 15 degrees C/min to 230 degrees C (hold 5 min)
- Inlet: Heater at 250 degrees C, Split ratio 2:1
- Carrier Gas: Ar at 2.0 mL/min (constant flow mode)
- Injection Volume: 1 microliter

S3. Short-Term (3 h) Bulk Electrolysis Evaluation of Ag-Spray-Coated Cu Electrode

To evaluate the physical and catalytic reproducibility of the Ag nanoparticle spray-coating method, independent short-term (3 h) bulk electrolysis evaluations were performed under identical operational conditions using separately fabricated Ag-spray-coated Cu electrodes at a constant current density of −25 mA/cm2. The time courses of Faradaic efficiencies for major gaseous products (CH_4, C_2H_4, CO, and H_2) and the operational electrode potential over 3 h of continuous electrolysis are shown in Figure S1.

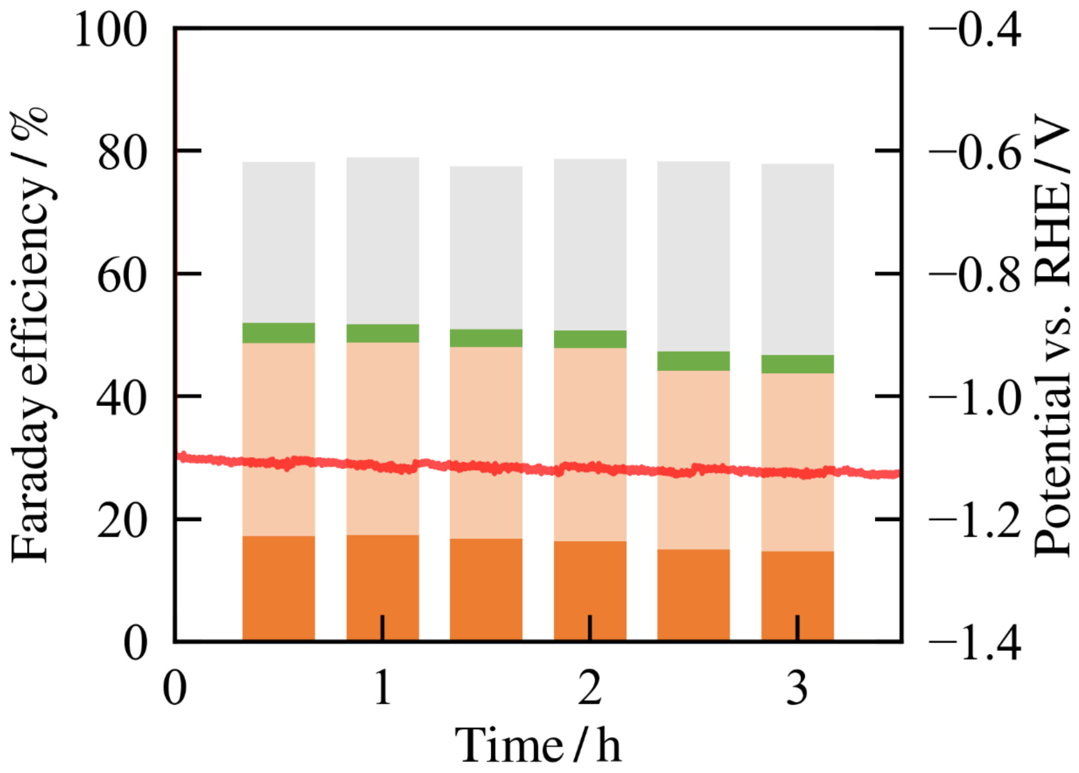

Figure S1. Faradaic efficiencies of gaseous products (CH4, C2H4, CO, and H2) and cell voltage during 3 h of continuous CO2 reduction using the Ag-spray-coated Cu electrode at a constant current density of −10 mA/cm2.
